# High Expression of CLEC11A Predicts Favorable Prognosis in Acute Myeloid Leukemia

**DOI:** 10.3389/fonc.2021.608932

**Published:** 2021-03-02

**Authors:** Chengliang Yin, Junyan Zhang, Wei Guan, Liping Dou, Yuchen Liu, Ming Shen, Xiaodong Jia, Lu Xu, Rilige Wu, Yan Li

**Affiliations:** ^1^ Medical Big Data Research Center, Medical Innovation Research Division of Chinese People's Liberation Army General Hospital, Beijing, China; ^2^ Faculty of Medicine, Macau University of Science and Technology, Macau, China; ^3^ National Engineering Laboratory for Medical Big Data Application Technology, Chinese People's Liberation Army General Hospital, Beijing, China; ^4^ Department of Hematology, Chinese People's Liberation Army General Hospital, Beijing, China; ^5^ Research Center for Translational Medicine Laboratory, Medical Innovation Research Division of Chinese People's Liberation Army General Hospital, Beijing, China; ^6^ Hepatobiliary Surgery Center, The Fifth Medical Center of Chinese People's Liberation Army General Hospital, Beijing, China; ^7^ Department of Hematology, Peking University, Third Hospital, Beijing, China

**Keywords:** CLEC11A, acute myeloid leukemia, prognosis, biomarker, methylation, expression

## Abstract

**Background:**

Acute myeloid leukemia (AML) is a heterogeneous disease of the hematopoietic system, for which identification of novel molecular markers is potentially important for clinical prognosis and is an urgent need for treatment optimization.

**Methods:**

We selected C-type lectin domain family 11, member A (CLEC11A) for study *via* several public databases, comparing expression among a variety of tumors and normal samples as well as different organs and tissues. To investigated the relationship between CLEC11A expression and clinical characteristics, we derived an AML cohort from The Cancer Genome Atlas (TCGA); we also investigated the Bloodspot and HemaExplorer databases. The Kaplan-Meier method and log-rank test were used to evaluate the associations between CLEC11A mRNA expression, as well as DNA methylation, and overall survival (OS), event-free survival (EFS), and relapse-free survival (RFS). DNA methylation levels of CLEC11A from our own 28 *de novo* AML patients were assessed and related to chemotherapeutic outcomes. Bioinformatics analysis of CLEC11A was carried out using public databases.

**Results:**

Multiple public databases revealed that CLEC11A expression was higher in leukemia. The TCGA data revealed that high CLEC11A expression was linked with favorable prognosis (OS *p*-value = 2e-04; EFS *p*-value = 6e-04), which was validated in GSE6891 (OS *p*-value = 0; EFS *p*-value = 0; RFS *p*-value = 2e-03). Methylation of CLEC11A was negatively associated with CLEC11A expression, and high CLEC11A methylation level group was linked to poorer prognosis (OS *p*-value = 1e-02; EFS *p*-value = 2e-02). Meanwhile, CLEC11A hypermethylation was associated with poor induction remission rate and dismal survival. Bioinformatic analysis also showed that CLEC11A was an up-regulated gene in leukemogenesis.

**Conclusion:**

CLEC11A may be used as a prognostic biomarker, and could do benefit for AML patients by providing precise treatment indications, and its unique gene pattern should aid in further understanding the heterogeneous AML mechanisms.

## Introduction 

AML is a heterogeneous clonal disorder featured by the accumulation of somatic genetic alterations in hematopoietic progenitor cells, changing normal mechanisms of self-renewal (1, 2), cell proliferation, and differentiation (3) with a highly variable prognosis and a high mortality rate (4, 5). At present, diagnosis and prognosis of AML are based primarily on morphology, immunophenotype (6), chromosome karyotype (7), and molecular features (8) according to the NCCN Guidelines (9).

Clinical and genetic prognostic markers are crucial in the evaluation of AML patients and in guiding rational management (2, 5). The detection of select cytogenetic markers serves as the strongest predictive marker for determining prognostic subgroups in AML (10, 11). Recent discoveries also have shed light on the important role of dysregulated transcription and epigenetic mechanisms in the pathogenesis of AML (12–15). Several studies have reported that the expression of different panels of genes that include CLEC11A is associated with clinical outcome in patients with AML (16, 17). In our previous study on the prognosis of AML, a panel of 12 methylated genes that included CLEC11A was found with hyper-differentially promoter methylated regions for prognosis in AML (18). CLEC11A (also known as P47, SCGF, LSLCL, and CLECSF3) is located on human chromosome 19 (19q13.33) and encodes a member of the C-type lectin superfamily (https://www.ncbi.nlm.nih.gov/gene/6320). Previous studies revealed that aberrant methylation and expression of CLEC11A were linked with the prognosis of pancreatic cancer, lung cancer, and thoracic malignant tumor (19, 20). CLEC11A has high expression in bone marrow and is a stem cell growth factor functioning within the hematopoietic microenvironment, and playing an important role in the control of cellular differentiation (21). High expression of CLEC11A in plasma has been used as an indicator of hematopoietic recovery and is associated with hemoglobin levels in patients following bone marrow transplantation (22, 23). The CLEC11A expression level is associated with clinical and biological prognostic outcomes in childhood acute lymphoblastic leukemia (16, 24, 25). Previous studies have also validated CLEC11A as a novel regulator and candidate therapeutic target of multiple myeloma SET domain (MMSET)-related myeloma (16, 26, 27). These outcomes indicated that CLEC11A plays a vital role in hematopoietic regulation. However, the precise prognostic value of CLEC11A in AML still remains unclear. This study shows that CLEC11A is a prognostic biomarker that may serve as an indicator for clinical recommendations.

## Methods

### Public Databases

Public data on CLEC11A expression, methylation, and related patient clinical data were selected from several databases as follows: (1) Oncomine database (www.oncomine.org): The Oncomine database is an online platform including microarrays from various sources, usually cancer-related, and contains many “multi-arrays.” It can figure out gene expression signatures, gene set modules, and so on (28). (2) Tumor IMmune Estimation Resource (TIMER) (https://cistrome.shinyapps.io/timer/): TIMER is a comprehensive resource for systematic analysis of immune infiltrates across diverse cancer types, whose DiffExp module allows users to study the differential expression between tumor and adjacent normal tissues for any gene of interest across all TCGA tumors. Users can identify genes that are up- or downregulated in the tumors compared to normal tissues for each cancer type, as displayed in gray columns when normal data are available (29). (3) The Human Protein Atlas (HPA) (https://www.proteinatlas.org): HPA has provided an integrated platform for researchers to study protein localization and expression in human organs, tissues, and cells. The centerpiece of the HPA is its unique antibody collection for mapping the entire human proteome by immunohistochemistry and immunocytochemistry (30). (4) Gene Expression Omnibus (GEO; http://www.ncbi.nlm.nih.gov/geo/): Gene Expression Omnibus is a public repository including microarray and sequencing genomic dataset (31). In this study, we studied a GEO cohort (GSE9476, 38 healthy donors, 26 AML patients), and the relationship between CLEC11A expression and the Kaplan-Meier survival analysis in AML analyzed *via* the TCGA data was further validated *via* a GEO database (GSE6891); (5) The Cancer Genome Atlas (TCGA) (https://tcga-data.nci.nih.gov/tcga/): The Cancer Genome Atlas is a public-funded landmark program that seeks to catalog and discover major cancer-causing genomic alterations to create a comprehensive “atlas” of cancer genomic profiles (32). In outline, we obtained gene expression data of mRNA and promoter methylation from TCGA, severally, which contributed 163 non-M3 *de novo* AML patients for mRNA gene expression, including 149 cases with both mRNA expression and DNA methylation data. We sorted 163 samples by mRNA expression values and subdivided into low and high groups by CLEC11A expression median value. The low-group contained 82 samples and the high-group contained 81 samples. The relationship of CLEC11A expression, methylation, and survival was analyzed. (6) BloodSpot (http://servers.binf.ku.dk/bloodspot/): BloodSpot is a database of gene expression profiles and transcriptional programs for healthy and malignant hematopoiesis. The function of BloodSpot is to provide an expression plot of genes in healthy and cancerous hematopoietic cells at specific differentiation stages (33). HemaExplorer database (http://servers.binf.ku.dk/hemaexplorer): The HemaExplorer is a curated database of processed mRNA Gene expression profiles (GEPs) that provides an easy display of gene expression in hematopoietic cells (34).

### MethylC-Capture Sequencing of AML Patients

In this study, a total of 28 patients with *de novo* AML were enrolled in the study, visiting our hematology department from August 2014 to December 2017. For these patients, clinical information and DNA samples from bone marrow (BM) were collected and obtained. DNA methylation of CLEC11A was detected by methylC-capture sequencing (MCC-Seq) and analyzed with the remission rate of chemotherapy in patients. The definition of induction remission followed the revised recommendations of the International Working Group (35), and complete remission (CR) meant BM blasts <5%, blasts without Auer rods, the absence of extramedullary disease, a neutrophil count >1 × 109/L and a platelet count ≥100 × 109/L. More detailed methods about the MCC-Seq and chemotherapy regimens are provided in our previous study (18). The research programme was authorized by the ethics committee of the General Hospital of Chinese People’s Liberation Army.

### Bioinformatics Analysis of CLEC11A

Between low and high CLEC11A mRNA expression groups, hierarchical clustering based on RNA expression levels were carried out and visualized by heat map and volcano plots using the data from TCGA. Gene ontology (GO) annotations of functional enrichment of overlapped genes (top twenty modules) was developed by Metascape (http://metascape.org/). The genes interacting with CLEC11A were also investigated in STRING (https://string-db.org/).

### Statistical Analyses

The prognostic endpoints used in this study were overall survival (OS), event-free survival (EFS), and relapse-free survival (RFS). OS was defined as the time from date of diagnosis to death; EFS was defined as the time from date of diagnosis to removal from the study caused by the absence of complete remission, relapse, or death; and RFS was identified from the first complete response (CR) to relapse, the last follow-up, or death. In the Oncomine database, the statistical cutoff values were a *p*-value threshold of 1 × 10^-4^, a fold-change of 2, and a gene rank threshold of top 10%. In TIMER, the distributions of gene expression levels were displayed using box plots, with statistical significance of differential expression evaluated using the Wilcoxon test (with a statistical cutoff of *p* < 0.05). For the correlation between CLEC11A expression levels and clinical characteristics, we applied Fisher’s exact test and the Wilcoxon rank-sum test to categorical and continuous variables, respectively. The Kaplan-Meier method and log-rank test were performed to evaluate the relationship between CLEC11A expression and prognosis (OS, EFS, and RFS). Multivariate analyses were performed by Cox proportional hazards models joining together several potentially independent prognostic factors (i.e., patient’s age, WBC, mutations of NPM1, CEBPA, TP53, or FLT3). The OS and EFS of the 163 samples from TCGA database were analyzed using the “forestplot” R package to present the p-value, HR, and 95% CI of each variable. R software was conducted to plot the nomogram and predict the 1-year, 2-year, and 3-year OS and EFS nomogram. In this article, a two-sided *p*-value < 0.05 was considered statistically significant.

## Results

### High Expression of CLEC11A in AML

**Figure 1:**
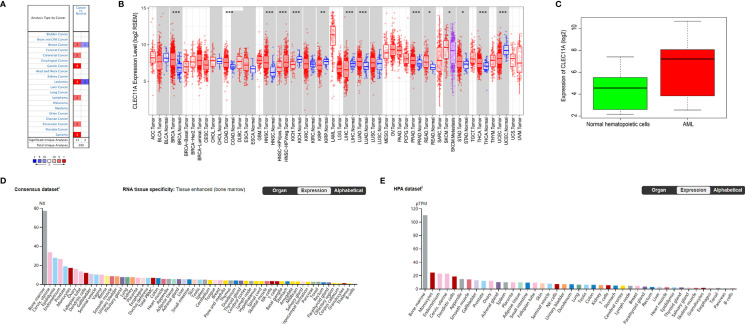
**(A)** CLEC11A expression levels in different tumors and normal tissues of multiple cancer types in the Oncomine database. **(B)** The differential expression between the tumor and adjacent normal tissues for CLEC11A across all TCGA tumors *via* TIMER. **(C)** Comparing CLEC11A expressions in AML and normal hematopoietic cells in GSE9476. **(D, E)** RNA expression overview of CLEC11A in different organs in the Consensus dataset and HPA dataset. *, p<0.05. **, p<0.01. ***, p<0.001.

We used the Oncomine database to analyze the variations in CLEC11A mRNA levels in different tumors and normal tissues of multiple cancer types. This analysis showed that CLEC11A expression was higher in breast, colorectal, gastric, pancreatic cancers and leukemia, lymphoma, sarcoma tumors than in normal tissues (**Figure 1A**).

To further explore CLEC11A expression in human cancers, we examined CLEC11A expression in TCGA. The differential expression between the tumor and normal tissues for CLEC11A is shown in **Figure 1B** in all TCGA tumors. It was obvious that CLEC11A expression in AML was the highest among all the tumors and normal tissues. Furthermore, a cohort (GEO database, GSE9476) of AML and normal hematopoietic cells showed that CLEC11A expression was much higher in AML than in normal hematopoietic cells (*p* = 4.78e-04) (**Figure 1C**).

We also investigated the data in Consensus dataset and HPA dataset, and found that CLEC11A expression level in bone marrow was the highest among all the tissues (**Figures 1D, E**).

### Differences of Clinical Features Between Low CLEC11A Expression Group and High Group

With TCGA data, we investigated the differences of some clinical characteristics between low CLEC11A expression group and high group. It revealed that FAB subtype M0, mutated genes RUNX1 and TP53, complex karyotype, and risk status all had statistically significant *p*-values (**Table 1**).

**Table 1:** Patients’ characteristics in the cohort of 163 TCGA patients according to CLEC11A expression level.

Clinicopathological characteristic	Low-CLEC11A group (n = 82)	High-CLEC11A group (n = 81)	P-value
Gender, male no (%)	52	54	9.33e-01
Age, median y (range)	62 (18–88)	55 (21–81)	6.13e-02
<60	39 (contains 60)	53 (contains 60)	3.21e-02
>60	43	28	3.21e-02
WBC count, median ×10^9^/L (range)	15 (0.6–297.4)	29.4 (1.5–171.9)	4.68e-01
BM blasts, median (range)	68.5 (32–99)	73 (30–100)	6.61e-02
PB blasts, median (range)	38.5 (0–98)	40 (0–97)	5.98e-01
FAB subtype, no			
M0	13	3	1.91e-02
M1	23	21	8.98e-01
M2	17	23	3.40e-01
M4	17	18	9.67e-01
M5	7	14	1.52e-01
M6	1	1	1.00e+00
M7	3	0	2.48e-01
Fusion gene, no			
BCR-ABL1	2	1	1.00e+00
PML-RARA	0	0	NA
MYH11-CBFB	2	9	5.82e-02
NUP98-NSD1	1	2	9.91e-01
MLL-PTD	3	6	4.81e-01
Mutated gene, no			
FLT3	21	23	8.23e-01
NPM1	23	25	8.24e-01
CEBPA	6	7	9.82e-01
IDH1	6	10	4.15e-01
IDH2	11	6	3.18e-01
RUNX1	13	4	4.30e-02
DNMT3A	26	17	1.69e-01
TP53	14	1	1.25e-03
KIT	2	5	4.30e-01
ASXL1	3	0	2.48e-01
TET2	8	8	1.00e+00
High ERG, no	44	37	3.89e-01
High BAALC, no	41	40	1.00e+00
High MN1, no	46	35	1.37e-01
High WT1, no	2	8	9.85e-02
Normal karyotype, no	39	40	9.40e-01
Complex karyotype, no	19	4	1.82e-03
Risk status, no			7.63e-04
Good-risk	2	16	1.05e-03
Intermediate-risk	51	49	9.50e-01
Poor-risk	27	15	5.44e-02

**Figure 2:**
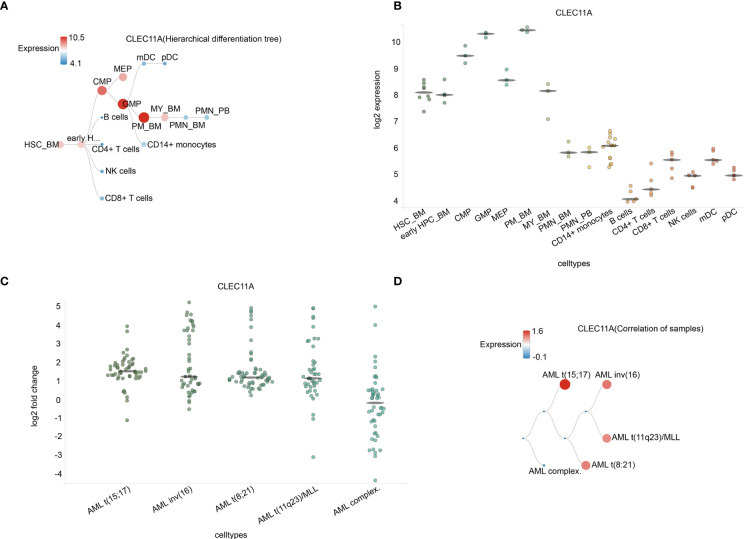
**(A)** Hierarchical difference tree about cell types with CLEC11A1 expression. **(B)** The expression of CLEC11A in a microarray in cell types. Horizontal lines represent the median expression for each class of cells. **(C)** Correlation of samples in different AML subtypes. **(D)** The expression of CLEC11A in different AML subtypes. Horizontal lines represent the median expression for each class of cell types. HSC_BM, Hematopoietic stem cells from bone marrow; early HPC_BM, Hematopoietic progenitor cells from bone marrow; CMP, Common myeloid progenitor cell; GMP, Granulocyte monocyte progenitors; MEP, Megakaryocyte-erythroid progenitor cell; PM_BM, Promyelocyte from bone marrow; MY_BM, Myelocyte from bone marrow; PMN_BM, Polymorphonuclear cells from bone marrow; PMN_PB, Polymorphonuclear cells from peripheral blood; B cells, CD19+ B cells; NK cells, CD56+ natural killer cells; mDC, CD11c+ myeloid dendritic cells; pDC, CD123+ plasmacytoid dendritic cells.

Further, BloodSpot and HemaExplorer database analysis found that the highest expression of CLEC11A was discovered in hematopoietic progenitor cells from granulocyte monocyte progenitors (GMP) and hematopoietic stem cells from promyelocyte from bone marrow (PM_BM) (**Figures 2A, B**). The results also showed significant differences in CLEC11A expression among various subtypes of AML (**Figures 2C, D**).

### High CLEC11A Expression Is Associated With Favorable Survival in AML

**Figure 3:**
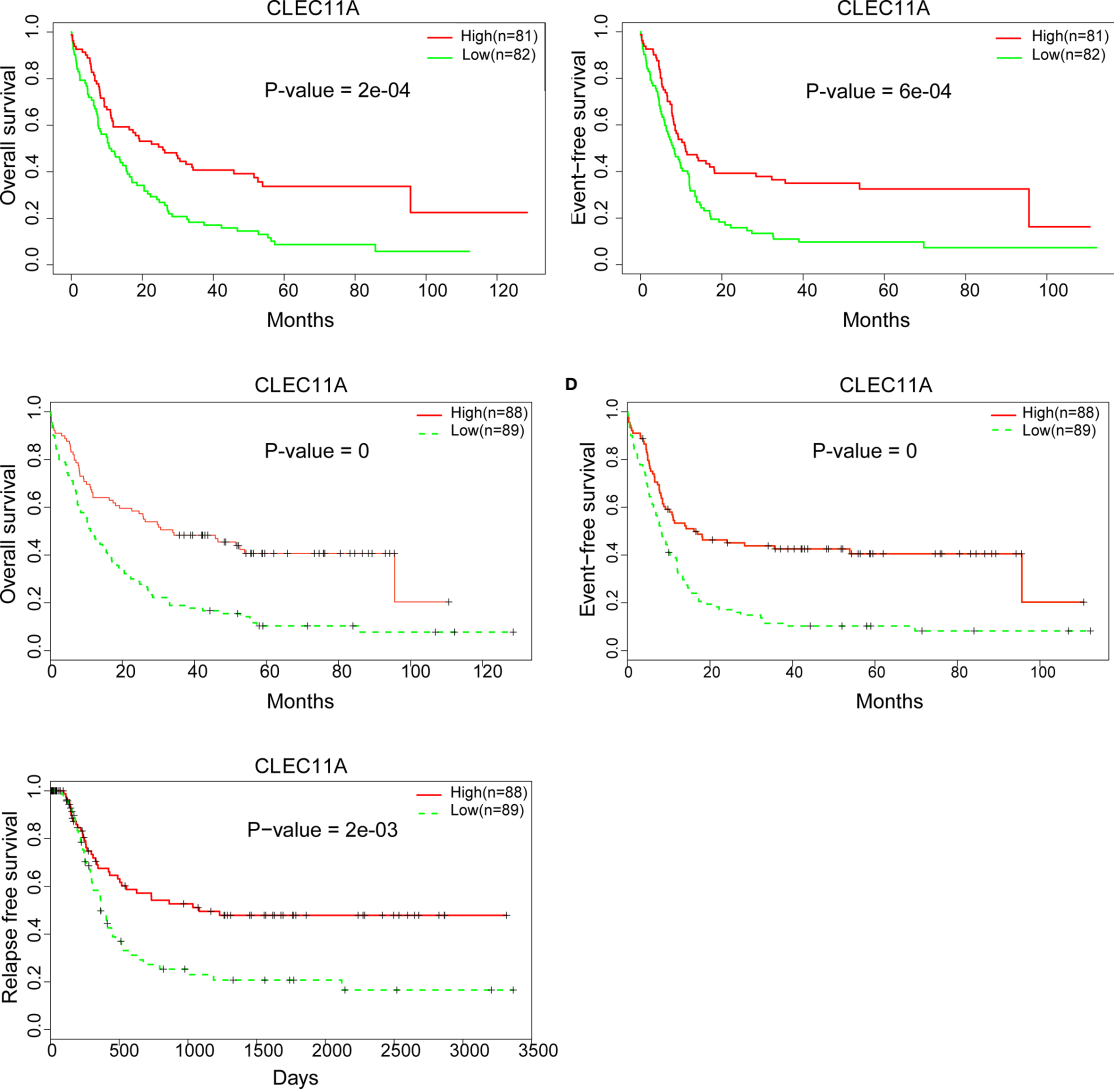
**(A)** Comparing the low and high expression of CLEC11A with OS in the TCGA dataset. **(B)** Comparing the low and high expression of CLEC11A with EFS in TCGA dataset. **(C)** Comparing the low and high expression of CLEC11A with OS in GSE6891. **(D)** Comparing the low and high expression of CLEC11A with EFS in GSE6891. **(E)** Comparing the low and high expression of CLEC11A with RFS in GSE6891.

Patient survival comparison was conducted between low and high CLEC11A expression groups in different datasets. The results showed that high CLEC11A expression was linked to better prognosis (OS *p*-value = 2e-04; EFS *p*-value = 6e-04) (**Figures 3A, B**). Meanwhile, the same conclusion was reached in the validation dataset (GSE6891, OS p-value = 0; EFS p-value = 0; RFS p-value = 2e-03) (**Figures 3C–E**).

**Figure 4:**
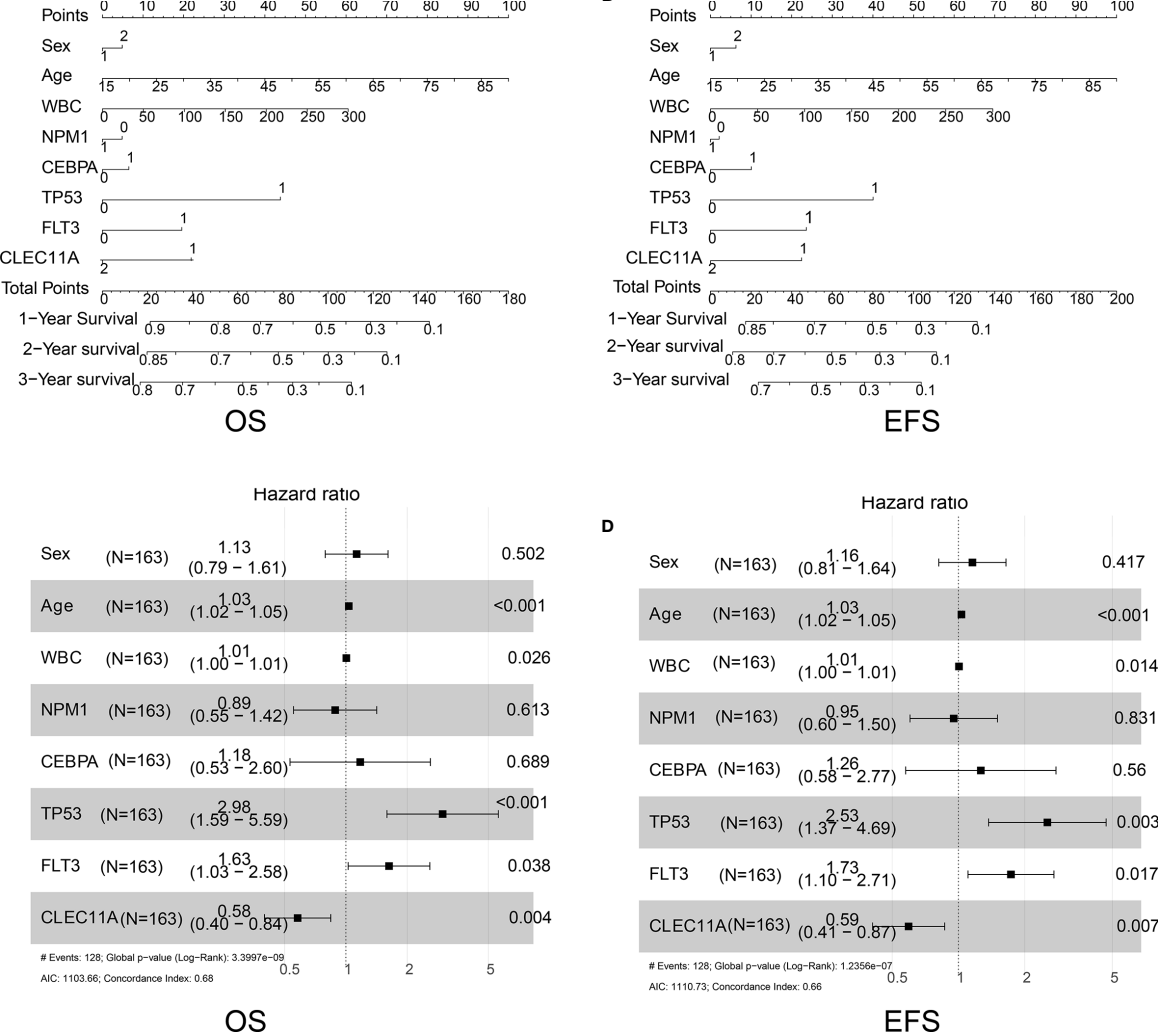
**(A)** Nomogram of OS in TCGA dataset. **(B)** Nomogram of EFS in TCGA dataset. **(C)** Forest plots of OS in TCGA dataset. **(D)** Forest plots of EFS in TCGA dataset.

We further constructed forest plots and nomogram of OS and EFS from the 163 samples together with independent prognostic factors (i.e., patient age, WBC, mutations of NPM1, CEBPA, TP53, or FLT3, and expression of CLEC11A). The nomogram quantitatively and intuitively displayed the probability of 1-year, 2-year, and 3-year OS and EFS (**Figures 4A, B**). Forest plots of OS and EFS showed that the confidence interval of the CLEC11A effective quantity was to the left of the invalid line (**Figures 4C, D**), indicating that high CLEC11A expression was a protective factor. This conclusion was in agreement with the Cox regression analysis (**Figures 3A–C**).

### CLEC11A DNA Methylation Level Is Associated With AML Induction Remission Rate and Survival

**Figure 5:**
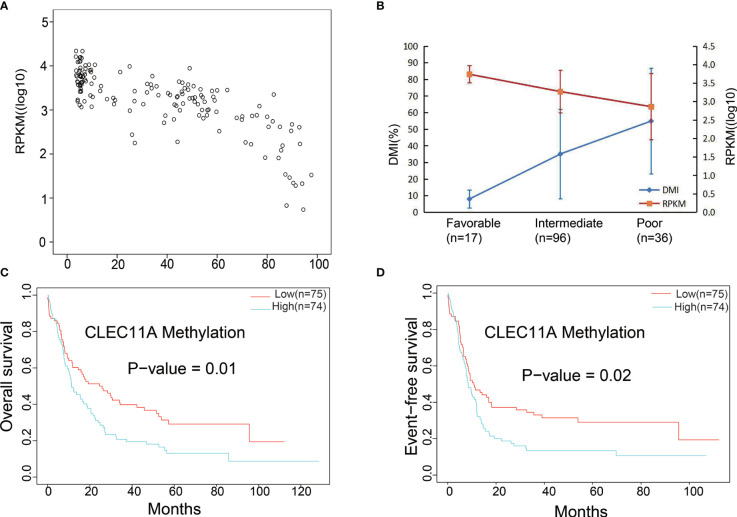
**(A)** Significant negative correlation between mRNA expression and DNA methylation data. **(B)** Gene expression levels and DNA methylation differences between each risk stratification were integrated to observe any relationship between methylation and expression. DMI (%), DNA methylation levels; RPKM, gene expression levels. **(C)** Comparing the low and high DNA methylation levels with OS in TCGA dataset. **(D)** Comparing the low and high DNA methylation levels with EFS in TCGA dataset.

Subsequently, the relationship between the CLEC11A methylation level, namely the DNA methylation index (DMI) and mRNA expression (RPKM), was assessed using the DNA methylation and RNA Seq data for the 149 TCGA patients with *de novo* AML. This revealed a significant negative correlation between DMI and RPKM (r = −0.778, *p* < 0.001, **Figure 5A**). The DMI of CLEC11A was shown to significantly increase cytogenetic risk stratifications (*p* < 0.001), whereas the RPKM significantly decreased (*p* < 0.001) (**Figure 5B**).

The prognosis was compared between low and high CLEC11A DNA methylation levels groups. The results showed that high CLEC11A methylation levels group was linked to poorer prognosis (OS *p*-value = 1e-02; EFS *p*-value = 2e-02) (**Figures 5C, D**).

A total of 28 *de novo* AML patients in our center underwent retrospective detection of DNA methylation level of CLEC11A in bone marrow samples (33). The mean DMI of CLEC11A for patients in CR was less than that with none response (NR) (34.25 ± 5.88% *vs.* 46.30 ± 5.85%, *p* = 0.159). For the sake of further estimating the relationship between the regimen and CR, 28 patients were divided into a hypomethylation group (n = 14) and a hypermethylation group (n = 14) according to the median CLEC1A methylation level. In the 14 high-risk patients with hypermethylation, the CR rate of DCAG (including hypomethylating agents, decitabine) group (7/11, 63.6%) was better than that of the standard “7 + 3” group (1/3, 33.3%) (*p =* 0.538). In the 14 low-risk patients with hypomethylation, the CR rate of DCAG group was 75% (6/8) and that of the standard “7 + 3” group was 66.7% (4/6) (*p =* 1.000). These results suggested that CLEC11A hypermethylation was associated with a poor induction remission rate, but without a significant difference (8/14, 57.1% *vs.* 10/14, 71.4%, *p* = 0.695); these patients may benefit from a regimen including hypomethylating agents. The median OS for the high DMI and low DMI groups were 14.93 months and 23.8 months (*p =* 0.049), respectively, while the median DFS were 10.97 months and not defined (*p =* 0.395), respectively.

### Heat Map, Volcano Plots, and Functional Enrichment

**Figure 6:**
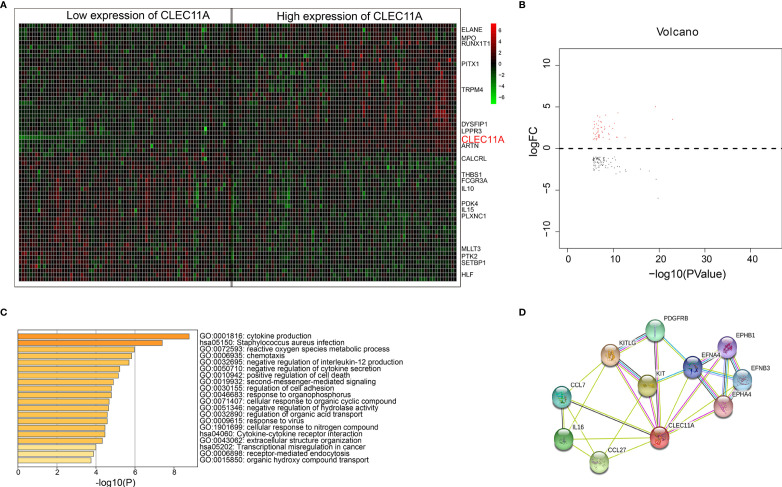
**(A)** Heat map for genes expression differential analysis between low and high CLEC11A expression of TCGA dataset. **(B)** Volcano plots for genes expression differential analysis between low and high groups of TCGA dataset. **(C)** Bar graph of enriched terms across enriched genes between low and high groups of the TCGA dataset, colored by *p* values. **(D)** Genes interacted with CLEC11A in STRING.

The analysis about CLEC11A-associated gene expression differential was performed to investigate the biological role of CLEC11A in the pathogenesis of leukemia by comparing the RNA expression levels of CLEC11A low expression group and high expression group. There were 207 upregulated and 179 downregulated genes associated with CLEC11A expression, both of which were presented in the expression heat map and volcano plots (**Figures 6A, B**). CLEC11A was seen in the heat map as an upregulated gene (**Figure 6A**). Functional enrichment showed that cytokine production and the negative regulation of cytokine secretion were relevant to CLEC11A (**Figure 6C**). We also analyzed the genes interacting with CLEC11A in STRING (**Figure 6D**).

AML is a heterogeneous clonal disorder featured by the accumulation of somatic genetic alterations in hematopoietic progenitor cells, changing normal mechanisms

## Discussion

AML is a heterogeneous clonal disorder featured by a clonal proliferation derived from primitive hematopoietic stem cells or progenitor cells (36). Immature malignant cells and fewer differentiated red blood cells, platelets, and white blood cells could be caused by abnormal differentiation of myeloid cells (36). Relapse remains the primary cause of treatment failure and mortality for AML patients (37). The clinical and genomic diversity of AML results in much of the difficulty in establishing a standard of care for relapsed AML (38). New management strategies are urgently needed for AML. Molecularly targeted agents will be a standard approach in induction and consolidation and serve as maintenance therapy after consolidation (39). Molecular testing plays a major role in AML (40) and is routinely performed as part of the diagnostic work-up. Our study provided direct evidence that high expression of CLEC11A predicted a favorable prognosis for AML. Firstly, CLEC11A expression in AML was obviously the highest among the multiple malignancies in TCGA *via* TIMER and significantly higher in AML tumors than normal tissues in GSE9476 (**Figures 1B, C**). Meanwhile, CLEC11A expression level in bone marrow was the highest among all the tissues in Consensus dataset and HPA dataset (**Figure 1D**). All these supported that CLEC11A was a significantly overexpressed gene in AML.

As for the clinical characteristics, patients with low CLEC11A expression were notably more than those with high CLEC11A expression in FAB M0 subtype compared with other subtypes, based on the TCGA cohorts (**Table 1**). Meanwhile, CLEC11A expression was statistically significantly associated with the mutated genes RUNX1 and TP53, complex karyotype, and risk status (**Table 1**). The BloodSpot and HemaExplorer database analyses found that the highest expression of CLEC11A was discovered in hematopoietic progenitor cells from granulocyte monocyte progenitors (GMP) and hematopoietic stem cells from promyelocyte from bone marrow (PM_BM), suggesting that CLEC11A was closely related to myeloid hematopoietic cells proliferation and differentiation (**Figures 2A, B**). These results demonstrated that CLEC11A was an early hematopoietic stimulator that might play an important role in the hematopoietic microenvironment of bone marrow (4, 21).

Several researches have studied on the expression and relationship of different gene panels with clinical outcomes in patients with AML (12, 13). Although CLEC11A was mentioned in these studies, the data used for analysis were only from TCGA datasets, and only the relationship between expression and clinical outcome was analyzed. Due to the different methods of analysis, the two studies obtained different gene panels and the former included six genes (*TREML2*, *SLC7A11*, *NLRP2*, *DDIT4*, *LSP*, and *CLEC11A*), while the latter included eight genes (*TET3*, *S100A4*, *BATF*, *CLEC11A*, *PTP4A3*, *SPATS2L*, *SDHA*, and *ATOX1*). Neither study focused on CLEC11A. Further, to better understand the correlation between CLEC11A expression and the prognosis of AML, the prognostic value of CLEC11A was confirmed in the independent group. A high level of CLEC11A expression was shown to be correlated with favorable OS and EFS in TCGA dataset (**Figures 3A, B**) and favorable RFS in GSE6891 (**Figure 3C**). Then we drew nomogram and forest plots of OS and EFS from the 163 samples of TCGA data (**Figures 4A–D**). This study demonstrated that CLEC11A was related to prognosis as a tumor suppressor gene. Moreover, in multivariable analyses including some known prognostic factors, high expression of CLEC11A was an independent factor for favorable OS and EFS (**Figures 4C, D**). Additionally, the nomogram showed a dramatic performance and clinical applicability (**Figures 4A, B**). All these results prove CLEC11A to be a potential biomarker, which might contribute to the precise prognosis and stratification of NCCN cytogenetically group in AML.

Moreover, CLEC11A DNA methylation also affected the levels of gene expression, and the methylation levels rose with increasing hazard ratio (HR) and poorer prognosis (**Figures 5A, B**). The application of demethylation drugs to inhibit the methylation of the CLEC11A gene is likely to be useful in treatment of AML. Additionally, DNA methylation was affected by the demethylating drug decitabine, and patients with higher CLEC11A methylation would be more suitable for DCAG therapy. Therefore, such a biomarker could be used to monitor cancer therapy with demethylating drugs that have shown promising results for AML.

The bioinformatics analysis indicated that CLEC11A was an upregulated gene (heat map, **Figure 6A**). Previous research showed that CLEC11A has a variety of biological activities and is mainly involved in cell proliferation and differentiation. Functional enrichment showed that cytokine production and the negative regulation of cytokine secretion were relevant to CLEC11A. Genes interacting with CLEC11A were also shown in STRING (**Figure 6D**). This study demonstrated that CLEC11A is an upregulated gene and might be crucial in the progress of leukemia.

The major limitation of the current study is that the results were mainly based on bioinformatics analysis of data in public databases. In the present study, we focused on the prognostic value of CLEC11A. We found and reported a phenomenon that high expression of CLEC11A predicts favorable prognosis in AML. Furthermore, the relationship between DNA methylation level and mRNA expression of CLEC11A was assessed, and the DNA methylation levels of CLEC11A of our own 28 *de novo* AML patients were detected and analyzed with chemotherapy. The relationship between methylation, expression, and prognosis has been initially presented by integrating the methylation, expression, survival, and chemotherapeutic efficacy, and it should be sufficient to draw a conclusion of the prognostic value of CLEC11A. We are also interested in the potential biological mechanism, and the relevant experiments are under investigation in our laboratory to verify the biological functions of CLEC11A. There are not enough results yet, so the experimental results are not given in this study and will be reported in future work.

## Conclusions

In summary, our study provides evidence that CLEC11A expression is high in AML, and low CLEC11A expression and high DNA methylation were associated with poor prognosis. We draw the conclusion that CLEC11A is a prognostic biomarker in AML. We also found that CLEC11A was an upregulated gene in AML. We hoped that CLEC11A’s peculiar gene patterns will help us to further understand the heterogeneity mechanism of AML. It might be used to predict the AML prognosis and could guide the precise treatment in the future for AML patients.

## Data Availability Statement

The original contributions presented in the study are included in the article/supplementary material. Further inquiries can be directed to the corresponding author.

## Author Contributions

YL and CLY designed the study. CLY, JYZ and WG collected and interpreted the data. CLY and JYZ drafted the manuscript. YL, WG, LPD, YCL, MS, XDJ, LX and RLGW provided the expert consultations and clinical suggestions. YL and CLY revised the manuscript. All authors reviewed the final version of the manuscript. All authors contributed to the article and approved the submitted version.

## Funding

This study was supported by National Natural Science Foundation of China (81800135 and 81902495), Medical Big Data and AI R&D Project of Chinese PLA General Hospital (2019MBD-001 and 2019MBD-025), National Key Research and Development Program of China (2017YFC0908400 and 2017YFC0908401).

## Conflict of Interest

The authors declare that the research was conducted in the absence of any commercial or financial relationships that could be construed as a potential conflict of interest.
